# Spatial distribution of malaria in Peninsular Malaysia from 2000 to 2009

**DOI:** 10.1186/1756-3305-7-186

**Published:** 2014-04-15

**Authors:** Haridah Alias, Johari Surin, Rohela Mahmud, Aziz Shafie, Junaidden Mohd Zin, Mahadzir Mohamad Nor, Ahmad Shah Ibrahim, Christina Rundi

**Affiliations:** 1 Department of Parasitology, Faculty of Medicine, University of Malaya, 50603 Kuala Lumpur, Malaysia; 2 Department of Geography, Faculty of Arts & Social Sciences, University of Malaya, 50603 Kuala Lumpur, Malaysia; 3 Ministry of Health Malaysia, Kuala Lumpur, Malaysia

## Abstract

**Background:**

Malaria is still an endemic disease of public health importance in Malaysia. Populations at risk of contracting malaria includes indigenous people, traditional villagers, mobile ethnic groups and land scheme settlers, immigrants from malaria endemic countries as well as jungle workers and loggers. The predominant species are *Plasmodium falciparum* and *P. vivax*. An increasing number of *P. knowlesi* infections have also been encountered. The principal vectors in Peninsular Malaysia are *Anopheles maculatus* and *An. cracens*. This study aims to determine the changes in spatial distribution of malaria in Peninsular Malaysia from year 2000–2009.

**Methods:**

Data for the study was collected from Ministry of Health, Malaysia and was analysed using Geographic Information System (GIS).

**Results:**

Changes for a period of 10 years of malaria spatial distribution in 12 states of Peninsular Malaysia were documented and discussed. This is illustrated by digital mapping according to five variables; incidence rate (IR), fatality rate (FR), annual blood examination rate (ABER), annual parasite index (API) and slide positivity rate (SPR).

**Conclusion:**

There is a profound change in the spatial distribution of malaria within a 10-year period. This is evident from the digital mapping of the infection in Peninsular Malaysia.

## Background

Worldwide, there are 109 malaria endemic countries with an estimated 3.3 billion people at risk of malaria in 2010, out of which 1.2 billion are at high risk (>1 case/1000 population). Of the 1.2 billion, 37% reside in Southeast Asia [1]. Malaysia was highly endemic with malaria, and in 1892, nearly one third of all deaths in Penang (an island state on the north-west of Malaysia) were attributed to malaria. Introduction of a new drainage system scheme by one of the pioneer antimalaria workers, i.e. Malcolm Watson in Peninsular Malaysia in the early twentieth century, was recognised as an early control measure in the world, leading to great reduction of malaria cases [2].

**Figure 1:**
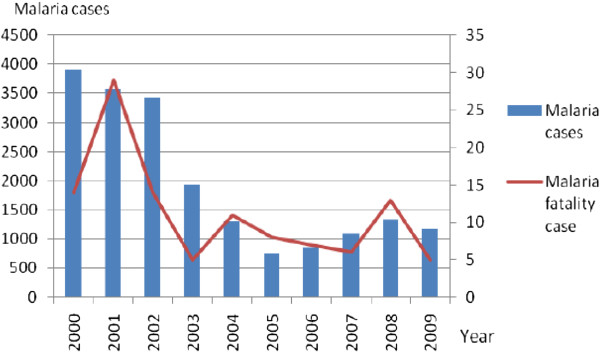
**Malaria cases and fatality cases in Peninsular Malaysia from 2000 to 2009.** Figure 1 shows the malaria cases and fatality cases reported in Peninsular Malaysia within ten years (2000 to 2009).

Being one of the major parasitic diseases in Malaysia, malaria affects indigenous people, traditional villagers, mobile ethnic groups and land scheme settlers, immigrants from malaria endemic countries as well as jungle workers and loggers [3,4]. The total number of malaria cases in Peninsular Malaysia is taking the downward trend from 3918 in 2000 to 757 in 2005. Since then, increasing trends were observed from 2006 with 852 cases, 2007 with 1106 cases and 2008 with 1342 cases. A total of 1175 cases were reported in 2009 (Figure 1) [Unpublished data from Vector Borne Disease Division Ministry of Health records]. Malaria was rarely reported in urban areas and Kuala Lumpur only reported 15 to 20 malaria cases from 2000 to 2007. However, in 2008 and 2009, an increased figure was observed with 27 and 49 cases respectively ([3], Unpublished data from Disease Control Division, Ministry of Health records). Recently, due to rapid influx of legal and illegal immigrant workers as well as a large number of tourist into this country, they became a new source of infection which resulted in the increase in detection of malaria cases in the Klang Valley area [5]. The total number of malaria cases among immigrants was 519 in 2006 and this increased to 690 in 2009 with 49.6% Indonesians, 17.1% Myanmarese, 11.2% Bangladeshis and 8.1% Thais. The remaining 14% of malaria cases were reported among immigrants from Pakistan, India, Nepal, Vietnam and Kampuchea (Unpublished data from Vector Borne Disease Division Ministry of Health records).

The highest number of malaria deaths in Peninsular Malaysia was reported to be 29 in 2001 and this figure decreased to 5 in 2009. The highest malaria incidence was recorded in the 20–39 years old age group ([3], Unpublished data from Disease Control Division Ministry of Health records). The younger age group who are actively working and highly mobile has increased the risk of being exposed to malaria infection, subsequently affecting the economic productivity due to work absenteeism. There were more males infected with malaria compared to females probably due to the former being involved in outdoor activities ([3,6], Unpublished data from Disease Control Division Ministry of Health records).

**Figure 2:**
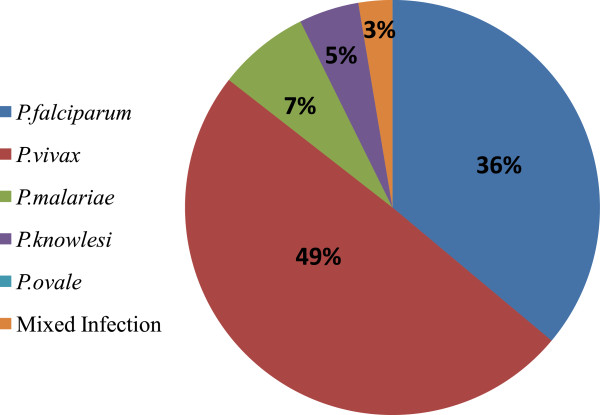
**Percentage of malaria cases by parasite species in 2009.** The percentage of malaria cases by five parasite species; *Plasmodium falciparum, P. vivax, P. malariae, P. ovale, P. knowlesi* and mixed infections.

Five Plasmodia species are known to infect humans, namely *Plasmodium falciparum, P. vivax, P. ovale, P. malariae* and *P. knowlesi* (simian malaria parasite). In Peninsular Malaysia, in year 2009, *P. vivax* is the predominant species detected with (49.5%), *P. falciparum* (36.1%), *P. malariae* (7.2%) and mixed infection (2.6%) (Figure 2). *P. knowlesi* had shown an increasing trend with 41 cases in 2008 to 55 cases in 2009 (Unpublished data from Disease Control Division, Ministry of Health records). Chloroquine and Fansidar resistance to *P. falciparum* has been reported in Peninsular Malaysia [7].

Important vectors involved in malaria transmission in Peninsular Malaysia are *Anopheles maculatus* and *An. cracens. An. maculatus* is the vector for human malaria whereas *An. cracens* is the vector for simian malaria caused by *P. knowlesi*[8]. With deforestation and environmental changes, new vectors have displaced the established vectors.

## Methods

### Study site

**Figure 3:**
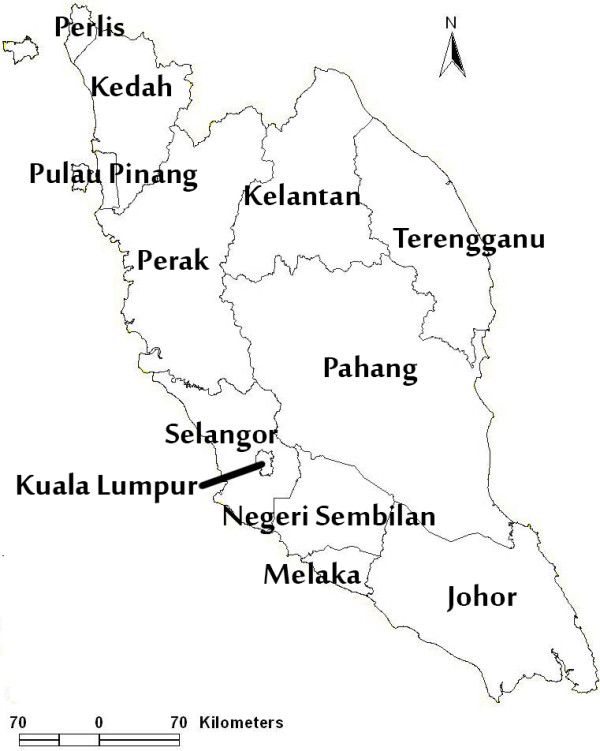
**Peninsular Malaysia map.** Figure 3 shows the 12 states in Peninsular Malaysia. Perlis, Kedah, Pulau Pinang and Perak situated at the northern part, Melaka, Negeri Sembilan, Federal Territory (Kuala Lumpur and Putrajaya) and Selangor at the central part, Pahang, Terengganu and Kelantan at east coast part and Johor at the southern part.

Peninsular Malaysia stretches between l°20′N to 6°40′N and from longitude 99°35′E to 104°20′E. It covers 131,598 km^2^ of geographical area. Peninsular Malaysia comprised 23.5 million people as per provisional data of 2012 census. Mean daily temperatures range from about 25°C to 28°C. The climate is equatorial, with rain from both the northeast (November to March) and southwest (May to August) monsoons. The rainfall ranges from a maximum of 5000 mm and a minimum of 1750 mm. The average maximum relative humidity of the air varies between 94% and 100%, typical of the humid tropics. Peninsular Malaysia consists of 12 states; Perlis, Kedah, Pulau Pinang, Perak, Melaka, Negeri Sembilan, Selangor, Johor, Pahang, Terengganu, Kelantan and Federal Territory (Kuala Lumpur and Putrajaya) (Figure 3) [9].

### Study design

The study was conducted using retrospective secondary data of malaria for the period of 2000 to 2009. We obtained all annual reports of malaria for the mentioned period from the Ministry of Health (MOH) Malaysia. Besides that, the data was also obtained from public health centers and local journals. For the updated population numbers, the data was taken from Department of Statistics (DOS) Malaysia which is available online. The approval from Malaysia National Health Institute (NIH) was successfully obtained in order to conduct the research in MOH. A second approval was given by MOH to allow the data collection process. Collected data was then entered in Microsoft Excel computer program. After proper data cleaning, the data was analyzed using Geographic Information System (GIS) to produce digital maps of incidence rate (IR), fatality rate (FR), annual blood examination rate (ABER), annual parasite index (API) and slide positivity rate (SPR).

## Results and discussion

Changes for a period of ten years of malaria spatial distribution in 12 states of Peninsular Malaysia are illustrated by digital mapping according to five parameters; IR, FR, ABER, API and SPR.

**Figure 4:**
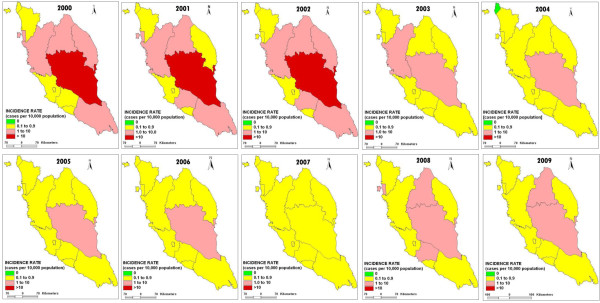
**The changes of malaria incidence rate (IR) in 12 states of Peninsular Malaysia from 2000 to 2009.** Figure 4 shows the changes of IR in 12 states of Peninsular Malaysia from 2000 to 2009.

The changes of malaria IR for 12 states in Peninsular Malaysia from 2000 to 2009 is graphically shown in Figure 4. Within 10 years duration, all states except Pahang reported malaria IR <10/10000 population. Pahang reported malaria IR >10/10000 population from 2000 until 2003. By 2000, Perlis, Kedah, Kuala Lumpur, Selangor and Melaka had virtually eliminated malaria ([3], Unpublished data from Disease Control Division, Ministry of Health records). The areas are stratifically colour coded based on the following criteria: (i) Red (malarious area): >10/10000 population, (ii) Yellow (malaria prone): <10/10000 population and (iii) Green (malaria free): no local transmission [10]. Distribution of insecticide treated nets (ITNs) and indoor residual spraying (IRS) to vulnerable areas successfully decreased the malaria cases in Peninsular Malaysia [3].

**Figure 5:**
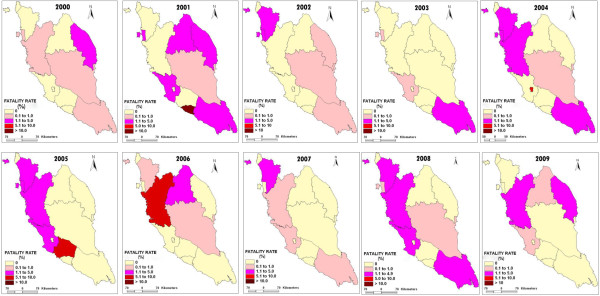
**The changes of malaria fatality rate (FR) in 12 states of Peninsular Malaysia from 2000 to 2009.** Figure 5 shows the changes of FR in 12 states of Peninsular Malaysia from 2000 to 2009.

Figure 5 shows the changes in FR for 12 states in Peninsular Malaysia from 2000 to 2009. There was no malaria fatality cases reported in Perlis. In Melaka, out of 15 confirmed malaria cases in 2001, two deaths were reported (13.33%). Kuala Lumpur only reported one fatality case (6.67%) from its total of 15 confirmed malaria cases in 2004. While in 2005, Negeri Sembilan reported one fatality case (9.09%) from its total of 11 confirmed malaria cases. In Kedah, Pulau Pinang, Perak, Pahang, Selangor, Kelantan, Johor and Terengganu the FR was <5% population within a 10 year period except for 2006 when Perak reported FR of 5.88% ([3], Unpublished data from Disease Control Division Ministry of Health records). Most of the deaths were caused by cerebral and complicated malaria. Factors that contributed to malaria deaths included delay in detecting malaria due to lack of suspicion of malaria when patient was first seen, failure in detecting severe and complicated malaria which led to improper treatment, delay in treating patient with IV Quinine because of no storage in District Hospital and cases were sometimes misdiagnosed as dengue, septicemia, typhoid and hepatitis [3].

**Figure 6:**
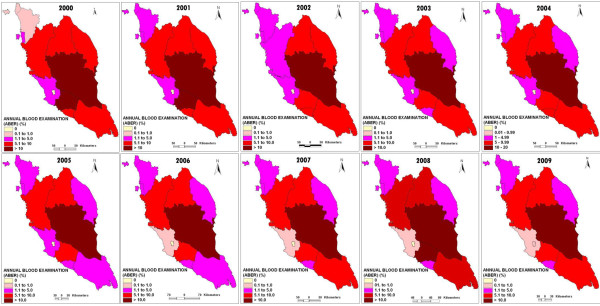
**The changes of Annual Blood Examination Rate (ABER) in 12 states of Peninsular Malaysia from 2000 to 2009.** Figure 6 shows the changes of ABER in 12 states of Peninsular Malaysia from 2000 to 2009.

Figure 6 shows the changes of ABER in 12 states of Peninsular Malaysia from 2000 to 2009. ABER reflects the efficiency and adequacy of case detection mechanisms. There are three mechanisms of malaria case detection in Peninsular Malaysia; passive case detection (PCD), active case detection (ACD) and mass blood survey (MBS) [3]. Within a 10 year period, the values of ABER in Pahang were constantly reported as more than 10%. Meanwhile, Negeri Sembilan reported ABER of >10%, twice (2000 and 2001). Perlis, Kedah, Pulau Pinang, Melaka, Selangor, Perak, Kelantan, Johor and Terengganu reported ABER values of less than 10% for a 10 year period. Kuala Lumpur reported the lowest ABER values of zero % from 2000 to 2008 that increased slightly to 0.1% in 2009 ([3], Unpublished data from Vector Borne Disease Division Ministry of Health records). The value of ABER was fixed at more than 10% per year under The National Malaria Elimination Program (NMEP) (Unpublished data from Disease Control Division, Ministry of Health records). By looking at the values, most of the states in Peninsular Malaysia still have an inadequate and inefficient case detection mechanism.

**Figure 7:**
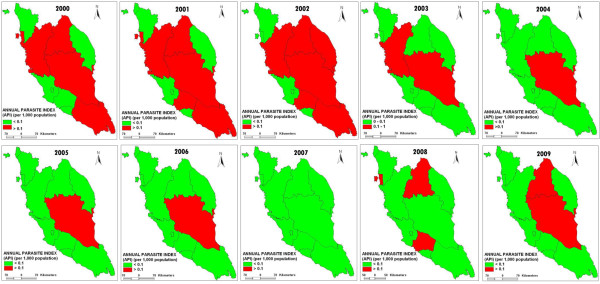
**The changes of Annual Parasitic Index (API) in 12 states of Peninsular Malaysia from 2000 to 2009.** Figure 7 shows the changes of API in 12 states of Peninsular Malaysia from 2000 to 2009.

The changes in API in 12 states in Peninsular Malaysia from 2000 to 2009 are shown in Figure 7. API depends on the adequacy of case detection mechanism i.e. ABER. If ABER is adequate, this parameter is the most important criterion to assess the progress of the eradication program. Perlis, Kedah, Selangor, Kuala Lumpur and Melaka reported API values of <0.1/1000 population for a 10 year period. Perak and Johor reported a decline in API values of >0.1/1000 population to <0.1/1000 population after the first four years. From 2000–2009, Pahang reported API values of >0.1/1000 population, except in 2008, the value is <0.1/1000 population. Pulau Pinang, Negeri Sembilan, Terengganu and Kelantan reported varied API values ranging from <0.1 and >0.1/1000 population ([3], Unpublished data from Disease Control Division, Ministry of Health records). Under NMEP, API value was fixed at <0.1/1000 population. To achieve the target, the area with high API values must be given priority and attention in terms of malaria control activities [11].

**Figure 8:**
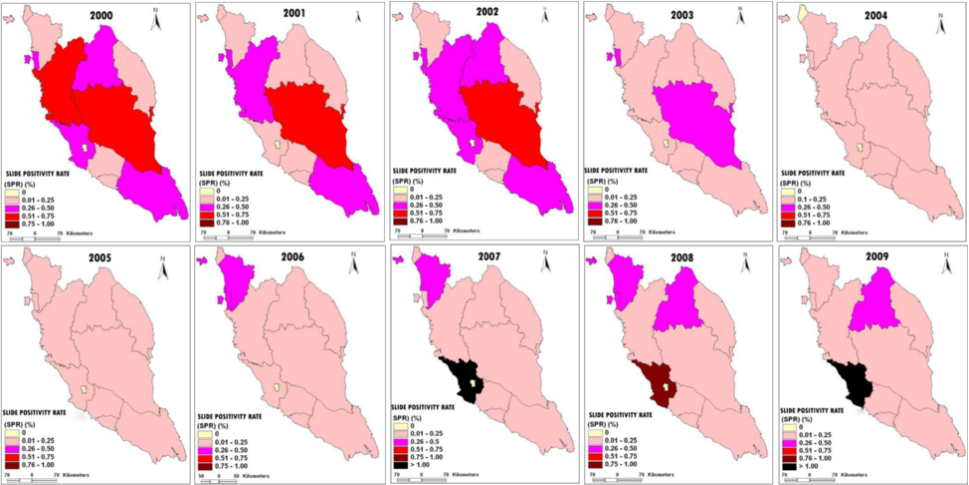
**The changes of Slide Positivity Rate (SPR) in 12 states of Peninsular Malaysia from 2000 to 2009.** Figure 8 shows the changes of SPR in 12 states of Peninsular Malaysia from 2000 to 2009.

Figure 8 shows the changes in SPR in 12 states in Peninsular Malaysia from 2000 to 2009. Whenever ABER is adequate, SPR is a dependable parameter for determining the progress of measures and gives information of parasitic load in the community. SPR measures the prevalence of malaria parasites among those who seek care and are examined in health facilities [11]. From 2000 to 2009, all states reported SPR values <1% except for Selangor and Kuala Lumpur. The SPR values were reported >1% (1.1%) in 2007 (Selangor) and 2009 (Selangor (1.04%) and Kuala Lumpur (4.98%) ([3], Unpublished data from Disease Control Division Ministry of Health records). In 2007, Selangor reported the highest malaria cases with 330 cases, mostly attributable to imported cases in immigrant workers. Within a10 year period (2000–2009), Kuala Lumpur recorded the highest malaria cases (49) in 2009, out of which 39 were imported cases [4]. WHO guidelines consider a country is ready to undergo transition from control to pre-elimination when the SPR value < 5% [11]. Malaysia is entering a pre-elimination stage and Peninsular Malaysia is targeted for malaria elimination by 2015 [4]. To achieve elimination, several strategies were implemented such as early case detection and prompt treatment, close monitoring of imported cases, residual spraying, usage of bed nets, environmental and anti larval management, monitoring of drug resistance and collaborating with the neighbouring countries [10].

## Conclusion

There is a profound change in the spatial distribution of malaria within a 10-year period. This is evident from the digital mapping of the infection in Peninsular Malaysia. The reduction in the number of malaria indigenous cases has been attributed to a successful Malaria Control Program (1980), which includes the increase in laboratory diagnostic capability, prompt treatment, nationwide implementation of insecticide treated bed nets and regular insecticide spraying.

## Competing interests

The authors declare that they do not have competing interests.

## Authors’ contributions

HA conceived the study and wrote the manuscript. HA, MMN and ASI performed the data collection. AS developed the GIS map. JS, RM, AS, JMC and CR revised the manuscript and gave approval of the version to be published. All authors read and approved the final manuscript.
